# Transpositional reactivation of the *Dart *transposon family in rice lines derived from introgressive hybridization with *Zizania latifolia*

**DOI:** 10.1186/1471-2229-10-190

**Published:** 2010-08-26

**Authors:** Ningning Wang, Hongyan Wang, Hui Wang, Di Zhang, Ying Wu, Xiufang Ou, Shuang Liu, Zhenying Dong, Bao Liu

**Affiliations:** 1 Key Laboratory of Molecular Epigenetics of MOE and Institute of Genetics & Cytology, Northeast Normal University, Changchun 130024, China; 2 Faculty of Life Science, Liaoning University, Shenyang 110036, China

## Abstract

**Background:**

It is widely recognized that interspecific hybridization may induce "genome shock", and lead to genetic and epigenetic instabilities in the resultant hybrids and/or backcrossed introgressants. A prominent component involved in the genome shock is reactivation of cryptic transposable elements (TEs) in the hybrid genome, which is often associated with alteration in the elements' epigenetic modifications like cytosine DNA methylation. We have previously reported that introgressants derived from hybridization between *Oryza sativa *(rice) and *Zizania latifolia *manifested substantial methylation re-patterning and rampant mobilization of two TEs, a *copia *retrotransposon *Tos17 *and a MITE *mPing*. It was not known however whether other types of TEs had also been transpositionally reactivated in these introgressants, their relevance to alteration in cytosine methylation, and their impact on expression of adjacent cellular genes.

**Results:**

We document in this study that the *Dart *TE family was transpositionally reactivated followed by stabilization in all three studied introgressants (RZ1, RZ2 and RZ35) derived from introgressive hybridization between rice (cv. Matsumae) and *Z. latifolia*, while the TEs remained quiescent in the recipient rice genome. Transposon-display (TD) and sequencing verified the element's mobility and mapped the excisions and re-insertions to the rice chromosomes. Methylation-sensitive Southern blotting showed that the *Dart *TEs were heavily methylated along their entire length, and moderate alteration in cytosine methylation patterns occurred in the introgressants relative to their rice parental line. Real-time qRT-PCR quantification on the relative transcript abundance of six single-copy genes flanking the newly excised or inserted *Dart*-related TE copies indicated that whereas marked difference in the expression of all four genes in both tissues (leaf and root) were detected between the introgressants and their rice parental line under both normal and various stress conditions, the difference showed little association with the presence or absence of the newly mobilized *Dart-*related TEs.

**Conclusion:**

Introgressive hybridization has induced transpositional reactivation of the otherwise immobile *Dart*-related TEs in the parental rice line (cv. Matsumae), which was accompanied with a moderate alteration in the element's cytosine methylation. Significant difference in expression of the *Dart*-adjacent genes occurred between the introgressants and their rice parental line under both normal and various abiotic stress conditions, but the alteration in gene expression was not coupled with the TEs.

## Background

It is widely recognized that hybridization between genetically differentiated natural plant populations may cause structural genomic changes (e.g., via homoeologous or ectopic recombination) as well as perturbation of epigenetic state of the recipient genome (e.g., DNA methylation), and both may result in heritable phenotypic novelties [1-7]. These findings are consistent with Barbara McClintock's insight of "genome shock", which proposed that crossing of different organismal species may cause restructuring of the resultant hybrid genome, and which may represent a facet of adaptive response by plants under specific circumstances [8]. A major cause underlying the genomic shock symptom is transcriptional and transpositional reactivation of otherwise cryptic transposable elements (TEs) in the hybrid genome. The reactivation of TEs is often coupled with disruption of chromatin-based epigenetic controlling mechanisms in the hybrid genome, like loss or re-patterning of cytosine methylation and compromised targeting by small interference (si) RNAs [9-11]. Indeed, several studies in both animals and plants have provided compelling empirical evidence in support of the "TE-epigenetic" basis of genome shock [12-16].

At least circumstantial evidence has indicated that for the hybridization- associated genomic shock to occur, a symmetric hybrid genome is not a prerequisite; instead, introgression or integration of "foreign" chromatin or DNA segments via introgressive hybridization or other means (e.g., transgenic) might as well produce the "shocking" effects on the recipient genome [11]. For example, it was shown in cultured animal cells that random integration of pieces of foreign DNA can cause the host genome to undergo extensive and genome-wide alterations in cytosine methylation of both cellular genes and TE-related DNA repeats [17,18]. We have demonstrated that introgression of small amount of chromatin of *Zizania latifolia *(a distantly related species to *Oryza*) into rice has caused an array of genetic and epigenetic instabilities in the recipient rice genome [19,20], and in particular, rampant mobilization of a *copia *retrotransposon *Tos17 *and a MITE (*mPing*) [21]. Given the recent finding that the cellular controlling mechanisms for TE activity are likely individualized [22], it is interesting to explore whether TE reactivation in the rice-*Zizania *introgressants was confined to these two elements or other TEs also experienced reactivation.

The rice *Dart *transposon family belongs to the *hAT *superfamily of class II TEs, and which was first characterized by Fujino and colleagues [23]. *Dart *was found as transcriptionally active in several rice tissues [24]. Moreover, both *Dart *and its deletion-derivative called *nDart *can be transpositionally active in certain rice genotypes that harbour active *Dart*, even under normal growing conditions [25]. In addition, the element's activity was correlated with its cytosine methylation state, and epigenetically silenced *Dart *copies can be reactivated by 5-azacytidine treatment [25,26]. Apparently, except for *Tos17 *and *mPing*, the *Dart/nDart *represents another family of highly active TEs endogenous to the rice genome.

The aim of this study was to investigate (1) whether the *Dart *TE family was transpositionally reactivated in the same set of rice-*Zizania *introgressants that showed rampant mobilization *of Tos17 *and *mPing *[21]; (2) whether the element's activity was correlated with its cytosine methylation state; and (3) whether excision and reinsertion of the element copies impacted expression of their adjacent genes under normal or various abiotic stress conditions.

## Results

### The *Dart *transposon family was transpositionally reactivated in the rice-*Zizania *introgressants

**Figure 1:**
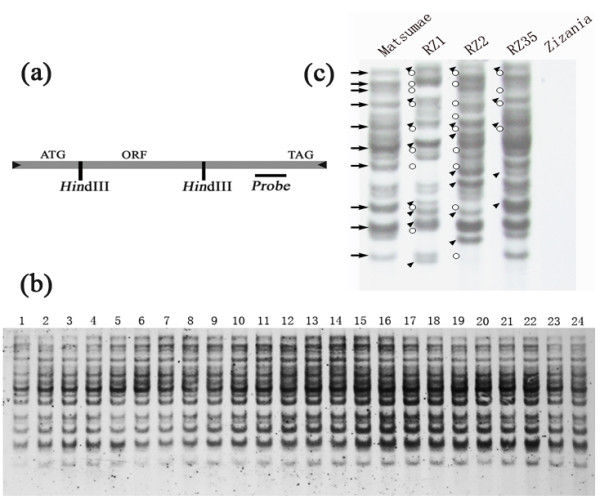
**Southern blot hybridization illustrating possible mobilization of the *Dart-*related TEs in three rice-*Zizania *introgressants and their immobility in the rice parental line**. (**a**) Diagram of a full-length copy of the *Dart*-related TEs showing the restriction site of the enzyme (*Hin*dIII) used, and the probe-targeting region (based on Fujino et al. 2005. *Mol Genet Genomics *273: 150-157). (**b**) Hybridization of the *Dart *probe to *Hin*dIII-digested genomic DNAs of 24 randomly chosen individual plants of the parental rice line Matsumae; the monomorphic pattern across the plants pointed to stability of *Dart*-related TEs in this rice cultivar. (**c**) Hybridization of the same probe to *Hin*dIII-digested genomic DNAs of three introgressants (RZ1, RZ2 and RZ35), their rice parental line (Matsumae) and the wild donor species, *Zizania latifolia*. The rice parental bands disappeared from one or more of the three introgressants (marked by circles) were indicated by arrows. Novel bands appeared *de novo *in the introgressants were denoted by arrowheads. No hybridization signal was detectable in *Z. latifolia*, indicating lack of a homologue of the *Dart*-related TEs in this wild species, and hence, none of the novel bands in the introgressants were due to direct introgression.

Based on the sequence of a full-length copy of *Dart1 *[23], we used the same pair of primers that should be specific to all conserved *Dart*-related elements to amplify a 296 bp fragment within the ORF region, designed by Fujino *et al. *[23]. The fragment was verified by sequencing, and then used as a probe for Southern blotting on *Hin*dIII-digested genomic DNAs of the three representative introgressants of rice-*Zizania *(RZ1, RZ2 and RZ35) together with their recipient rice parental line (Matsumae) and the donor species *Z. latifolia*. Since there are only two restriction site of *Hin*dIII within the full-length *Dart *(Figure 1a), Southern blotting with this enzyme-digest and a probe residing on one side of both of the restriction sites should enable a conservative estimation on the copy number of all conserved *Dart*-related elements and their possible transpositions or rearrangements. Because in certain rice genotypes that harbour intrinsically active *Dart *copies, the elements can be spontaneously transposing even under normal growing conditions [25], it was important to determine whether this was the case for cultivar Matsumae, the rice parental line for the introgressants. We thus first tested this possibility in 24 randomly selected individuals of three successive selfed-generations of Matsumae, and subjected the plants to the Southern blot analysis probed by the *Dart*-specific fragment. We found a uniform hybridization pattern for all the 24 plants (Figure 1b), indicating that Matsumae, as in most rice cultivars [23], did not contain an active *Dart*. In contrast, each of the three introgressants showed a dramatically changed hybridization pattern, and all were substantially different from that of their rice parental line Matsumae (Figure 1c). Because the donor species, *Z. latifolia*, did not contain a homologue of *Dart-*related element (no hybridization signal), the Southern blotting results suggest that most of the *Dart *copies were likely transpositionally reactivated in the rice-*Zizania *introgressants, and produced an array of excisions and reinsertions in the introgressants (Figure 1). Nonetheless, it should be noted that an alternative explanation that the changed hybridization patterns in the introgressants resulted from genomic rearrangements involving the *Dart*-related TEs could not be ruled out at this stage solely based on the Southern blot analysis.

**Figure 2:**
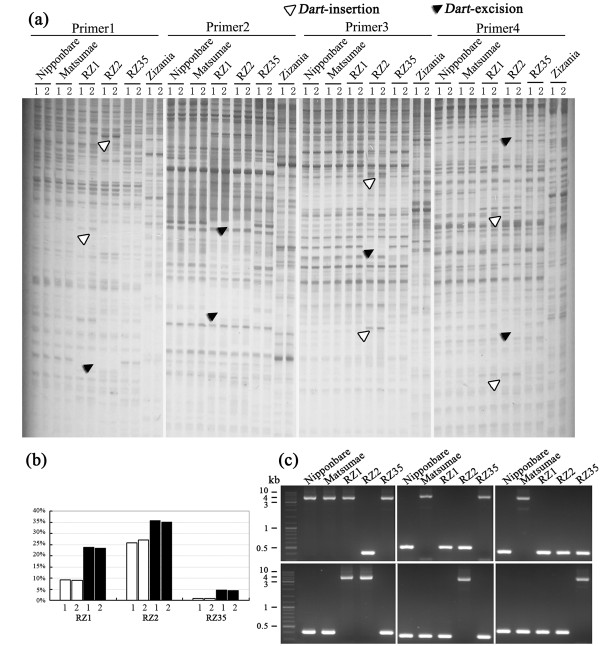
**Mobilization of the *Dart-*related TEs in three rice-*Zizania *introgressants revealed by transposon-display (TD) and validated by locus-specific PCR amplification**. (**a**) Exemplary profiles of *Dart*-specific TD. (**b**) Tabulated frequencies of excision and reinsertion of the element in each of the introgressants based on the TD-data. (**c**) Validation of the element transpositional activity by locus-specific PCR amplifications. The black and while triangles labelled in (**a**) denote excisions and reinsertions, respectively. Lanes 1 and 2 in (**a**) and (**c**) refer to TDs with the *iDart1-51*/*nDart1-3 *and *iDart1*s/*nDart1-101*s subgroup-specific primers (detailed in the manuscript text). The upper panel in (**c**) (from left to right) are examples of *Dart*-excisions that occurred in one (RZ2), two (RZ1 and RZ2) and all three (RZ1, RZ2 and RZ35) introgressants, which were detected with primers *Dart*-TDE-5, *Dart*-TDE-2 and *Dart*-TDE-3, respectively; the lower panel in (**c**) (from left to right) are examples of *Dart*-reinsertions that occurred in two (RZ1 and RZ2), one (RZ2) and one (RZ35) of the introgressants, respectively, which were detected with primers *Dart*-TDI-7, *Dart*-TDI-1 and *Dart*-TDI-27, respectively. Note that for a given locus, the fully sequenced standard rice cultivar Nipponbare, based on which the locus-specific primers were designed, may or may not contain the *Dart *copy.

To distinguish the two possibilities between transposition and rearrangement as a cause for the changed Southern blotting patterns of *Dart*-related TEs in the rice-*Zizania *introgressants, we resorted to the *Dart*-specific transposon-display (TD) developed by Tsugan *et al. *(2006) [25] to isolate possible excision and reinsertion events that likely had occurred in the introgressants relative to their rice parental line. The results of the TD profiles showed that both the patterns and total numbers of amplified bands were very similar between the *iDart1-51*/*nDart1-3 *and *iDart1*s/*nDart1-101*s subgroups with the primer pairs used, indicating they were mainly targeting to overlapping genomic regions (e.g., Figure 2a). An unexpected observation was that many bands were also amplified from the donor species, *Z. latifolia *(e.g., Figure 2a). Because there was no homologue for the *Dart*-related TEs in the *Zizania *genome both based on the Southern blotting results (Figure 1c) and on the *Dart-*specific PCR amplification using *Z. latifolia *genomic DNA as a template (data not shown), we suspect that these bands were not resulted from TD, but from the homo-amplification by the *Mse*I-adaptor primers in the silver-stained profiles, in which all amplicons were visible (in contrast to radioactively albelled primers). To test this, we sequenced 17 bands isolated from the *Z. latifolia *lanes; mainly those bands the positions of which were either identical or proximal to bands existing in the introgressant(s) but not in the rice parent were selected to maximize the likelihood of detecting possible *Zizania *introgression. We found that all the sequenced bands contained the same *Mse*I-adaptor primer at both ends, and none contained the terminus of *Dart*-related TE that should be expected for a *bona fide *TD band. This suggested that (1) the novel bands in the introgressant(s) were not likely due to direct introgression from the donor species, *Z. latifolia*; (2) there was probably no homologue of *Dart*-related TEs in *Z. latifolia*, consistent with the Southern blotting and *Dart*-specific PCR amplification results (Figure 1c). The tabulated *Dart*-TD results by the 18 primer pairs (see Additional file 1) enabled an gross estimate of the putative excision (loss of rice parental bands) and reinsertion (gain of novel bands) frequencies of 9% and 24% for RZ1, 26% and 35% for RZ2, and 1% and 5% for RZ35, respectively (Figure 2b). These results indicated that, first, more insertions than excisions had occurred in all three introgressants; second, there was marked difference in the transpositional activity of the *Dart*-related TEs among the three introgressants, with RZ2 showed the most and RZ35 the least activities.

Next, we cloned and sequenced a subset of 60 bands, each of which was variable in one or more of the introgressants relative to the corresponding position in the rice parental line (Matsumae) in the TD profiles. These variant bands included 30 putative excisions (present in Matsumae but absent from at least one of the introgressants) and 30 putative insertions (appeared *de novo *in one or more of the introgressants). We found that for each of the clones sequenced, the expected terminus encompassing the 19-bp terminal inverted repeat (TIR) of *Dart *was identified, indicating that these isolated bands were resulting from real TD rather than from homo-amplification by the adaptor primers. Further sequence analysis identified 15 and 21 of the variant TD bands as most likely resulted from excision and insertion events, respectively, because these bands contained the typical 8-bp target-site duplication (TSD) of *Dart *(Tables 1 and 2). Nonetheless, their authenticity as *bona fide *excisions and insertions rather than rearrangements entails further validation, as detailed below.

**Table 1:** Excision sites of *Dart*-related TEs identified by transposons-display (TD) (designated as Dart-TDE) in the rice-*Zizania *introgressants

Excision sites	**Excision position**^**§**^	Locus-specific primers (5'-3')	Excised from	Excision flanks
Dart-TDE-1	Chr.2 Position:11650578 Non-coding	For: tgcgcacaatcacctctatg Rev: cttgtggtgcaaccaaccta	RZ1, RZ2	acaatcacctctatg<Dart>aatgcacacactcat acaatcacctctatg---------aatgcacacactcat
Dart-TDE-2	Chr.4 Position: 2633169 Non-coding	For: gtgcgcgtaatgctagaaaa Rev: cagggagggagggattagag	RZ1, RZ2	acaatcacctctatg<Dart>aatgcacacactcat acaatcacctctatg---------aatgcacacactcat
Dart-TDE-3	Chr.7 Position: 20137929 Non-coding	For: cccccatacttaccgcatta Rev: cctaggtgttctgccgactc	RZ1, RZ2, RZ35	ttgtaaattaactcc<Dart>aaaccatctcctatc ttgtaaattaactcc---------aaaccatctcctatc
Dart-TDE-5	Chr.11 Position:16475838 Non-coding	For: tgagtgacgtgaagccaaag Rev: acagatcacggcagggttac	RZ2	aggtcggttaagcct<Dart>aagaaccacgaataa aggtcggttaagcct------aagaaccacgaataa
Dart-TDE-7	Chr.4 Position:18387453 Non-coding	For: cctctaggcacctccctttt Rev: caggagcaacaattgcatgt	RZ2	ctcccttttttttta<Dart>gaactaatgactttt ctccctttttttaa gaactaatgactttt
Dart-TDE-8	Chr.1 Position:18893612 Non-coding	For: tggtttggaggtcggttaag Rev: cacatgtcagccaaaaccac	RZ2	catcagattaagaaa<Dart>tccggtgaaaccatc catcagattaagaaa---------tccggtgaaaccatc
Dart-TDE-10	Chr.11 Position:22507932 Non-coding	For: gagaagagcacgggaagttg Rev: aactggctgttcgctcaagt	RZ2	taccgcattaaccac<Dart>ttaggtaggatacat taccgcattaaccac---------ttaggtaggatacat
Dart-TDE-11	Chr.8 Position: 15326441 Non-coding	For: tcgtgttcccaaattcacac Rev: catatatcccgcagaaaagca	RZ1, RZ2	cctccacctctaca<Dart>aaaatttctactgttc cctccacctctaca--------aaaatttctactgttc
Dart-TDE-15	Chr.3 Position: 20176647 Non-coding	For: aacgagagcaagggagatgaa Rev: ttaagccagggcaagtacacg	RZ2	tcctacgtcactg<Dart>ttgaggcgagccaaa tcctacgtcactg---------ttgaggcgagccaaa
Dart-TDE-16	Chr.9 Position: 21368076 Non-coding	For: gtgcatggattttgaccttta Rev: ctgtgctcacttcgctactacta	RZ1	atattgccatttaa<Dart>gtgtcatcgcctta atattgccatttaa--------gtgtcatcgcctta
Dart-TDE-19	Chr.10 Position: 7832680 Non-coding	For: ggtgtaacgattgctaaggcg Rev: agtggggggagagtaagatga	RZ2	tcttttttttacgca<Dart>tgcagaggtgacg tcttttttttacgca<Dart>tgcagaggtgacg
Dart-TDE-20	Chr.11 Position: 7910449 Os11g0247800 exon	For: agagttcttgccaaccatgc Rev: ggaagagggaaaaaccaagc	RZ1, RZ2	tctaatacctctag<Dart>gactgctttccacatg Tctaatacctctag--------gactgctttccacatg
Dart-TDE-21	Chr.7 Position: 16720991 Non-coding	For: cgatcgagaatttccgagac Rev: tggtctgttcgttgtccaaa	RZ1	tttcaccccctatat<Dart>tggtaccatcaattt Tttcaccccctatat--------tggtaccatcaattt
Dart-TDE-23	Chr.6 Position: 6273602 Non-coding	For: cttttgggctgtgatggagt Rev: ttaaggacgatgccaaaacc	RZ2	ttctgtccaccccta<Dart>gctggtatttatat ttctgtccaccccta--------gctggtatttatat
Dart-TDE-25	Chr.6 Position: 26631010 Non-coding	For: cctcggtttccattagca Rev: gtacggcctggcaagtga	RZ1, RZ2	ttttgtccaccccta<Dart>tctactcctagttgc Ttttgtccaccccta--------tctactcctagttgc

^§^Excisions refer to events occurred in one or more of the three introgressants (RZ1, RZ2 and RZ35). Naturally, the corresponding loci in Matsumae all contain a copy of Dart-related TE, whereas in Nipponbare only some of the loci contain the element, pointing to genotypic polymorphism with regard to presence/absence of the *Dart*-related TEs for a given genomic locus.

**Table 2:** *De novo *insertion sites of *Dart*-related TEs identified by transposons-display (TD) (designated as Dart-TDI) in the rice-*Zizania *introgressants

Insertion sites	Position of insertion sites	Locus-specific primers (5'-3')	Inserted into	TIR (5'-3')	TSD (5'-3')
Dart-TDI-1	Chr.3; position: 2726981; non-coding	For: tcacgcagtagatgccaaag Rev: gcacgtctccgtagctctct	RZ2	gcccatttggccacctcta	tgctagta
Dart-TDI-3	Chr.10; position:4241777; OSJNAb0015J03.2 exon	For: gtagagggctcaatcgtgga Rev: ctaaggtctcgaggcacacc	RZ1, RZ2	gcccatttggccacctcta	gcatgaag
Dart-TDI-4	Chr.5; position: 29238540; non-coding	For: gcccgtttggccacctctat Rev: tgtaaaatgaccagcgacga	RZ1, RZ2, RZ35	gcccatttggccacctcta	tgtggttg
Dart-TDI-5	Chr.5; position:14917879; non-coding	For: tacggttcccattgttttcc Rev: gggtgtgcacgatgttgtaa	RZ2	gcccatttggccacctcta	tacaatgt
Dart-TDI-7	Chr.12; position:12726861; non-coding	For: ttgttgttagttttgcgtgtaga Rev: gaaagcaggttggagaggtta	RZ1, RZ2	gcccatttggccacctcta	cgctagta
Dart-TDI-8	Chr.3; position:27833204; Os03g0699200 exon	For: taattaagttggaagtgggaca Rev: tttctgtaagattacaaccagaggt	RZ1, RZ2, RZ35	gcccatttggccacctcta	tggagtat
Dart-TDI-10	Chr.3; position:17730615; non-coding	For: ctttcgtaggcgaaaagtgc Rev: ctgcaaccacctgtctctga	RZ2	gcccatttggccacctcta	cgaagaac
Dart-TDI-11	Chr.4; position:638561; non-coding	For: catgaattgggtgccatgta Rev: ccccatagggtaggcaaaat	RZ1, RZ2, RZ35	gcccatttggccacctcta	tctgaatt
Dart-TDI-16	Chr.5; position:10010289; non-coding	For: gcccgtttggccacctctat Rev: ggtggaggacctgctcaata	RZ1, RZ2	gcccatttggccacctcta	ttcgacat
Dart-TDI-18	Chr.12; position:6156546; non-coding	For: tgagcacgcctagctcagta Rev: atgcacggcaactttctctt	RZ1, RZ2	gcccatttggccacctcta	cctctcaa
Dart-TDI-19	Chr.12; position:6345593; non-coding	For: gcccgtttggccacctctac Rev: acaaatggcctcctgtgttc	RZ1	gcccatttggccacctcta	caagcagc
Dart-TDI-20	Chr.12; position:21951729; non-coding	For: tccagccaaaccctgttc Rev: gctcgccagatgtcaggt	RZ1	gcccatttggccacctcta	cgtcggga
Dart-TDI-21	Chr.1; position:19308190; non-coding	For: tgctacagtagaagggcgtgta Rev: atgcacatctggtcttttgatg	RZ2	gcccatttggccacctcta	cacacgta
Dart-TDI-22	Chr.2; position:6211564; non-coding	For: ggatccgtttggatcagaga Rev: tgcagcagctgattcatacc	RZ2	gcccatttggccacctcta	ccaatatt
Dart-TDI-24	Chr.5; position:10749306; OSJNBa0037H03.12 intro	For: gagctgctcctgaaaaccac Rev: gaattttccttgccgtgtgt	RZ1, RZ2	gcccatttggccacctcta	tcatgttt
Dart-TDI-25	Chr.1; position:25299; non-coding	For: gtgccggagaatgatttgat Rev: atttccctcgatgcactgtc	RZ1, RZ2	gcccatttggccacctcta	tacgcagc
Dart-TDI-26	Chr.2; position:10207338; Os02g0277600 intro	For: gcccatttggccacctcta Rev: cgaatgagtgtccttgatcg	RZ1, RZ2	gcccatttggccacctcta	agcaaaac
Dart-TDI-27	Chr.12; position:23594158; Os12g0572000 3'UTR	For: gcccgtttggccacctctac Rev: agcaacccacagaacagctt	RZ35	gcccatttggccacctcta	ccaccctc
Dart-TDI-28	Chr.8; position:23713525; non-coding	For: gcccgtttggccacctctac Rev: tctgcggttgaaacaatgag	RZ2	gcccatttggccacctcta	cggctaac
Dart-TDI-29	Chr.7; position:20028374; non-coding	For: gcccgtttggccacctctat Rev: aaagtcaatggaaaggggaaa	RZ1, RZ2	gcccatttggccacctcta	tcaaaatc
Dart-TDI-30	Chr.4; position:25704503; Os04g0514800 exon	For: gcccatttggccacctcta Rev: ggcaatgcggttggtttc	RZ1, RZ2	gcccatttggccacctcta	cgctattc

### Validation and chromosomal location of the *Dart *excisions and insertions by locus-specific PCR amplification and sequencing in the rice-*Zizania *introgressants

For each of the putative excised and inserted loci, we designed locus-specific primer pairs specific to the flanks of the *Dart *elements based on the whole genome sequence of Nipponbare http://rgp.dna.affrc.go.jp (Tables 1 and 2). Note that the design of primers targeting the flanks did not entail that Nipponbare contains a copy of the *Dart-*related TE at each of the loci. PCR amplification by these locus-specific primers (e.g., Figure 2c) and sequencing of the amplicons verified that all the 15 excisions and 21 insertions were authentic, because the immediate contiguous flanks were intact for all the excisions and typical TSDs were identified for all the insertions (Tables 1 and 2). This locus-specific PCR amplification and sequencing results thus validated transpositional reactivation of the *Dart*-related elements in the rice-*Zizania *introgressants.

**Figure 3:**
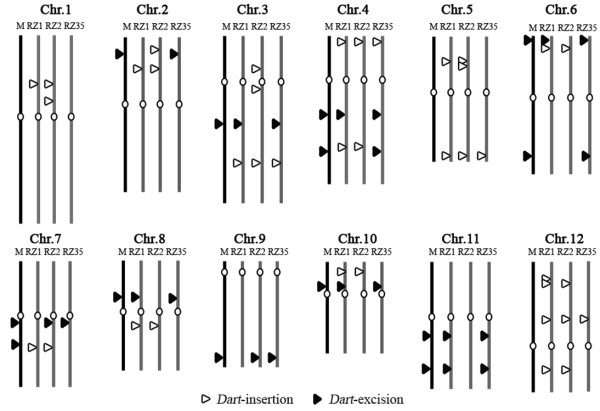
**Electronic mapping of the excisions and insertions of the *Dart*-related TEs in three rice-*Zizania *introgressants based on the whole-genome sequence of the standard rice cultivar Nipponbare http://rgp.dna.affrc.go.jp**. M denotes the rice parental line Matusumae. Excisions from the paretnal line and *de novo *insersions into the introgressant(s) were labelled.

We mapped all the 15 excisions and 21 insertions of *Dart *that occurred in the introgressants by aligning the identified flanking regions of each of the events against the whole genome sequence of Nipponbare. We found that the mobilized *Dart*-related elements mapped throughout the rice genome involving all the 12 chromosomes (Figure 3), suggesting that these seemingly random excisions and insertions by the *Dart*-related elements probably had not imposed an adverse effect on fitness of the introgressants, and hence, being selected for or neutrally retained during propagation of the plants via self-fertilization.

### Cytosine methylation states of the *Dart*-related elements in the rice-*Zizania *introgressants and their rice parental line

**Figure 4:**
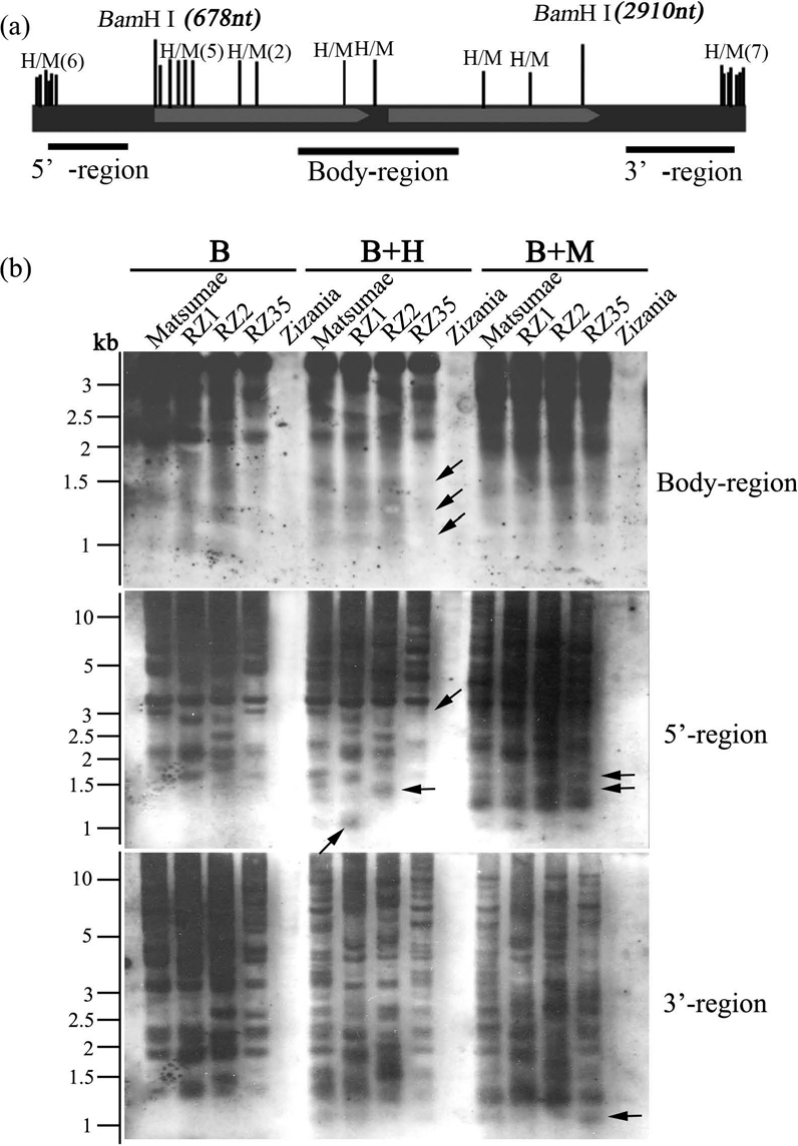
**Alteration in cytosine methylation at the 5'-CCGG sites within and immediately flanking the *Dart*-related TEs in three rice-*Zizania *introgressants, detected by methylation-sensitive Southern blot analysis**. (**a**) Restriction maps of enzymes *Bam*HI and *Hpa*II/*Msp*I for *Dart*-related TEs. The restriction sites for the enzyme (*Bam*HI) used to delineate *Dart*-related TEs and the pair of isoschizomers, *Hpa*II/*Msp*I (H/M), within the elements are indicated. Fragments to be used as probes that are respectively specific to the 5'-, 3'- and body-regions are denoted by solid bars. (**b**) Hybridization of each of the three probes (uppermost: the body-region, middle: the 5'-region, lowermost: the 3'-region) to a blot carrying single- or double-enzyme digested genomic DNA of the introgressants and their parental lines, rice (cv. Matsumae) and *Z. latifolia*. The enzymes used were indicated on the top of the blot: *Bam*HI (B), *Hpa*II (H) and *Msp*I (M). The altered bands indicative of methylation alterations in the introgressant(s) are arrowed.

Given our previous results that cytosine methylation of these rice-*Zizania *introgressants were substantially re-patterned relative to their rice parental line Matsumae [19], and the established frequent causal links between TE activity and its methylation state by numerous studies [9,10,22,24,27-30], it was interesting to test whether the transposition of *Dart *in the introgressants was accompanied with alteration in this epigenetic marker. Thus, we investigated the methylation state of the *Dart-*related elements in the introgressants *vs*. that in Matsumae. We first delineated the *Dart*-related elements by digestion with *Bam*HI, which had two restriction sites within *Dart1 *(and by extension all conserved copies of *Dart*-related TEs) at positions 678 nt and 2910 nt, respectively, and hence, in theory digestion with this enzyme would produce three fragments for each of the conserved *Dart *copies: the delineated internal body-region (2232 bp in length) and the 5'- and 3'-terminus together with their contiguous flanks (Figure 4a). We noted however that several major hybridization bands above the delineated 2232 bp body-region band were detected by the region-specific probe (Figure 4b, the body-region probe), indicating that either some of the *Dart-*related elements were not conserved at the *Bam*HI restriction site(s), or there had been insertions by related or unrelated sequences within the two restriction sites, and hence producing larger-sized fragments after the enzyme restriction. Nonetheless, those *Dart-*related elements that gave rise to the 2232 bp band were amenable to methylation analysis of the body-region, because there had been no apparent internal truncations, as detailed below.

**Figure 5:**
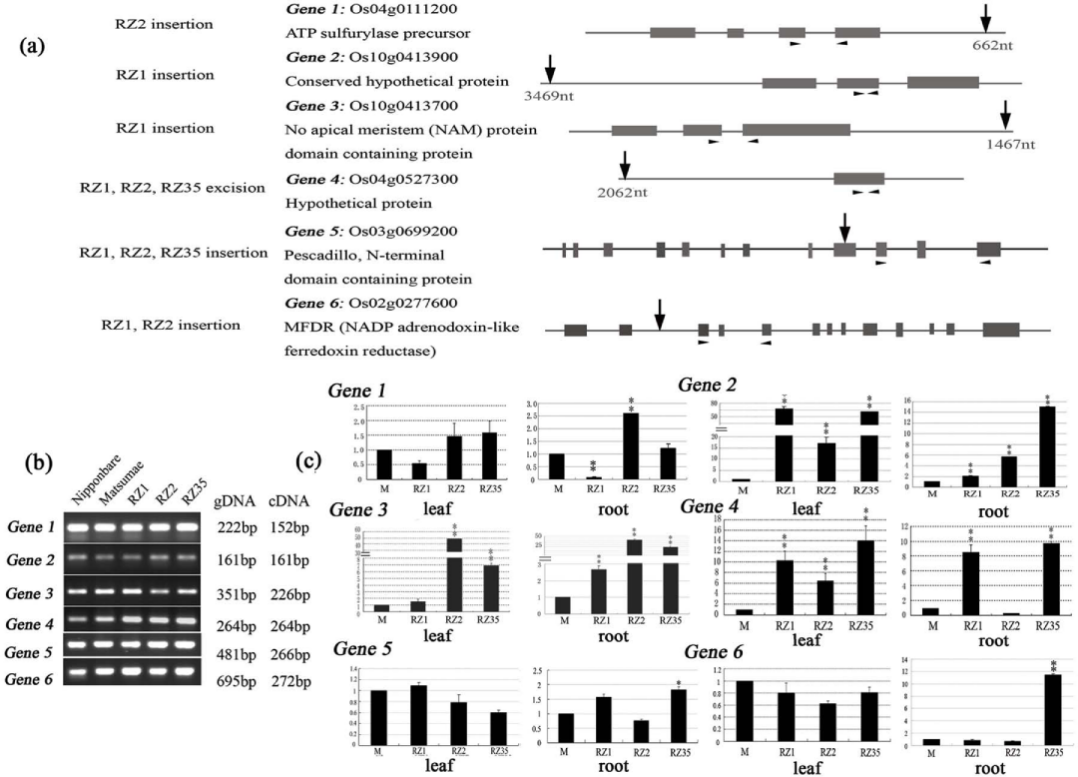
**Measurement of expression of six genes adjacent to the newly excised or inserted *Dart*-related TEs in two tissues (leaf and root) taken from the three rice-*Zizania *introgressant(s) and their rice parental line Matsumae under normal growing condition by real-time qRT-PCR analysis**. (**a**) Diagrams showing the excision or insertion positions (vertical arrows) of *Dart*-related TEs in each of the four genes, and positions (horizontal arrowheads) of the gene-specific primers. The grey rectangles denote exons for each gene. (**b**) Amplification of the six genes on genomic DNA as templates (20 ng each sample) from the introgressants and their rice parental line Matsumae. The standard cultivar Nipponbare was also included as an additional control. The near identical amplifications indicate lack of amplification bias by the designed primers. (**c**) Transcriptional expression of each of the six genes in the leaf and root tissues taken from the introgressants and their rice parental line, measured by real-time qRT-PCR. The relative abundance of transcripts (mean ± SD) for each of the studied genes was calculated upon normalization against a rice β-actin gene (Genbank accession X79378). * and ** denote statistical significance at the 0.05 and 0.01 levels, respectively.

We next performed the second round restriction by adding each of the pair of isoschizomers, *Hpa*II and *Msp*I, to the *Bam*HI-digests, and using probes specific to each of the three regions to assess their cytosine methylation state in the introgressants relative to Matsumae. We obtained the following results: (1) there had been no detectable internal truncations between the two *Bam*HI sites, as no clear, smaller-sized bands than the expected 2232 bp were detected in this enzyme digest (Figure 4b, the body-region probe); (2) the body-region of the conserved, *Dart*-related elements was heavily methylated particularly by ^m^CG in all the rice lines, introgressants and parental, as evidenced by the very similar hybridization patterns between *Bam*HI-digest and *Bam*HI+*Hap*II-digest (Figure 4b, the body-region probe), albeit there were 11 5'-CCGG sites within this region of *Dart*-related elements, and hence several smaller-sized bands would have been detected if the relevant 5'-CCGG sites were hypomethylated (Figure 4a); (3) the only clear difference in the methylation state of the introgressants relative to Matsumae was that one introgressant (RZ35) showed hypermethylation as evidenced by the disappearance of several smaller-sized bands (Figure 4b, the body-region probe; marked by arrows); (4) the 5' region of the *Dart*-related elements was also heavily methylated by both ^m^CG and ^m^CNG in the all the lines because multiple bands were detected (Figure 4b, the 5' probe), as otherwise we would have predominantly detected a band <678 bp in length (restricted by *Bam*HI at position 678 nt together with restriction by *Hpa*II/*Msp*I at one or more of the six 5'-CCGG sites at the 5'-terminus) (Figure 4a); (5) compared with Matsumae, CG demethylation in RZ1 and concomitant CG hypermethylation and CNG hypomethylation in RZ35 occurred in the 5'-region of *Dart*-related elements (Figure 4b, the 5' probe); (6) similar to the situation of 5'-region, the 3'-region of the *Dart*-related elements was also heavily methylated by both ^m^CG and ^m^CNG in all the lines (Figure 4b, the 3' probe), as otherwise we would have predominantly detected a band <656 bp in length (being restricted at position 2910 nt by *Bam*HI together with one or more of the seven 5'-CCGG sites by *Hpa*II/*Msp*I at the 3'-terminus) (Figure 4a); (7) compared with Matsumae, CG demethylation in RZ2 and CNG demethylation in RZ35 occurred in the 3'-region of the *Dart*-related elements (Figure 4b, the 3' probe); (8) with regard to the methylation state of the 5' and 3' flanks of the *Dart*-related elements, it was deducible that they had a substantially lower methylation level relative to the *Dart*-related elements *per se*, as evidenced by the multiple small-sized bands detected by the *Dart *5'- and 3'-region-specific probes (Figure 4b). Taken together the Southern blotting data of all three region-specific probes, it can be concluded that (1) the *Dart*-related TEs are heavily methylated throughout the entire length, but with their 5' and 3' flanks being relatively less methylated compared with the internal body-region, in all the rice lines studied; (2) to a moderate extent, methylation alteration including both hypo- and hypermethylation occurred in the introgressants relative to their rice parental line Matsumae.

### Limited impact by mobility of the *Dart*-related TEs on expression of their adjacent genes under normal and abiotic stress conditions

The seemingly random distribution of the excisions and insertions of the *Dart*-related TEs in the introgressants (Figure 3) raised the question as to whether their mobility had imposed any effect on expression of their neighbouring genes. To address this question, it was important to identify genes residing at unique-copy regions (ruling out possible confounding effects) neighbouring the newly mobilized insertion/excision sites of the *Dart*-related TEs; in addition, because we were interested in possible regulatory alterations rather than changes in the nucleotide sequence of the genes, we needed to select genes that would show identical amplification between the introgressants and their rice parental line if genomic DNA was used as templates. We thus extracted 10 kb of flanking genomic sequences (5 kb on each side of an excised or inserted *Dart*-related TE) from the whole genome sequence of Nipponbare and used them as queries for similarity search against the full-length cDNA database of rice http://cdna01.dna.affrc.go.jp/cDNA. We identified six genes meeting the criteria of unique-copy, encoding either known-function or hypothetical proteins, and identical amplification among all lines on genomic DNA templates (Figures 5a and 5b). Real-time qRT-PCR analysis was performed with primers specific to each of the six genes on cDNAs derived respectively from the leaf and root tissues from 21-day-old seedlings of the three introgressants and their rice parental line that were grown under normal growing conditions (Figure 5c). The results showed that all six genes exhibited significant difference in the transcript abundance in at least one tissue and one introgressant relative to Matsumae (Figure 5c). Specifically, (1) gene 1, to which a *Dart *copy being inserted 662 bp downstream in one introgressant (RZ2), showed significant down- and up-regulation, respectively, in the root tissue of RZ1 and RZ2; (2) gene 2, to which a *Dart *copy being inserted 3469 bp upstream in one introgressant (RZ1), showed significant up-regulation in both the leaf and root tissues (but more markedly in leaf) of all three introgressants though at variable degrees; (3) gene 3, to which a *Dart *copy being inserted 1467 bp downstream in one introgressant (RZ1), showed significant up-regulation in the leaf tissue of introgressants RZ2 and RZ35, and in the root tissue of all three introgressants at variable degrees; (4) gene 4, for which a *Dart *copy residing 2062 bp upstream being excised from all three introgressants (RZ1, RZ2 and RZ35), showed significant up-regulation in the leaf tissue of all three introgressants, and in the root tissue of introgressants RZ1 and RZ35 at variable degrees; (5) gene 5, to which a *Dart *copy being inserted into the 9^th ^exon in all three introgressants (RZ1, RZ2 and RZ35), showed significant up-regulation only in the root tissue of introgressant 35; (6) gene 6, to which a *Dart *copy being inserted into the 2^nd ^intron in two introgressants (RZ1 and RZ2), showed significant up-regulation only in the root tissue of introgressant 35. Albeit the dramatic changes (predominantly up-regulation) in transcription of all six studied genes in the introgressants relative to in the rice parental line, we noted that the changes were not associated with the presence or absence of the newly transposed *Dart*-related TEs under normal growing conditions.

**Figure 6:**
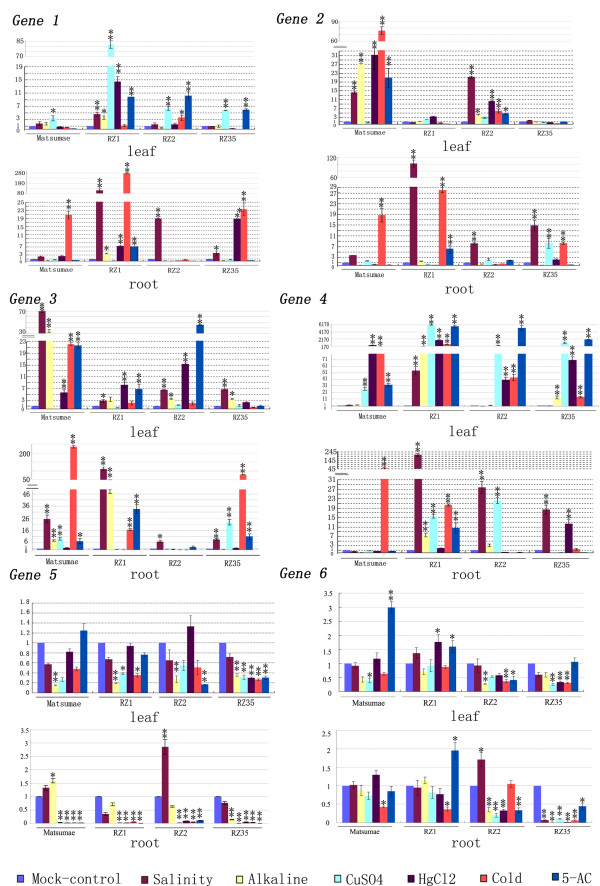
**Measurement of expression of the six genes adjacent to the newly excised or inserted *Dart*-related TEs in two tissues (leaf and root) taken from the three rice-*Zizania *introgressant(s) and their rice parental line Matsumae under the five abiotic stress conditions (salinity, alkaline, heavy metal -- CuSO4 and HgCl2, and cold) and after 5-azacytidine (5-AC) treatment by real-time qRT-PCR analysis**. The transcript measurements and statistical denotations are the same as in **Figure 5**.

We further investigated possible alteration in expression patterns of these six genes between the introgressants and their rice parental line under three kinds of abiotic stress conditions, salinity, alkaline, heavy metal (CuSO4 and HgCl2) and cold, which have been widely studied in rice [31]. We also included 5-azacytidine (5-AC) treatment to test for possible relevance of alteration in gene expression to changes in cytosine methylation of these genes. A general conclusion we were able to formulate based on the results was that four of the six genes (1 to 4) showed sharply differential response, both with regard to the kinds of treatments and to the extent of response, to each of the abiotic stresses as well as to 5-AC in the introgressants relative to their rice parental line in both tissues, but the rest two genes (5 and 6) showed grossly similar trend of responses to the treatments between the introgressants and their rice parental line in both tissues (Figure 6). For example, for gene1, significant alteration in its expression was detected only in one (CuSO4--upregulation) of the stress treatments in the leaf tissue and two treatments (alkaline--down-regulation and cold--up-regulation) in the root tissue, and 5-AC did not exert an effect, in Matsumae; in contrast, in the introgressants, this gene was responsive to most or all the stress treatments and also to 5-AC, and being predominantly up-regulated (Figure 6). An opposite situation was observed for gene 2 in the leaf tissue in that four of the five stress treatments (except CuSO4) as well as 5-AC induced significant up-regulation of this gene in Matsumae; in contrast, two of the introgressants (RZ1 and RZ35) did not respond to any of the treatments at all, and one introgressant (RZ2) responded to four of the five stress treatments and to 5-AC, but all responses were of lower extents (though the same trend, i.e., upregulation) relative to those in Matsumae (Figure 6). Gene 3 and gene 4 also exhibited sharply differential responses to at least some of the treatments between Matsumae and the introgressants for each of the tissues. Thus, a general conclusion for the first four genes (1 to 4) was that, in spite of the sharp difference between the introgressants and their rice parental line, they all represented up-regulation in response to the stresses and to 5-AC, probably because the basal expression level of these genes was relatively low in rice (Figure 6, genes 1 to 4), and which often belonged to genes with intrinsic methylation modifications [32]. In contrast, the rest two genes (5 and 6) showed relatively smaller difference between the introgressants and their rice parental line, and all were predominantly down-regulated in response to the stress treatments, and either no change or also down-regulation in response to 5-AC treatments, probably because the basal expression level of these two genes was relatively high in rice, and hence they were probably belong to intrinsically unmethylated genes [32]. Taken the data of all six genes together, it could be concluded that, similar to the situation of under normal growing condition, the expression patterns of theses genes in the introgressant(s) *vs*. their rice parental line under the various stress conditions and 5-AC treatment, though were substantially different, were again not coupled with the presence or absence of the newly mobilized *Dart*-related TEs. However, the fact that at least in one of the studied rice lines, expression of each of the four genes (1 to 4) was up-regulated by 5-AC treatment (Figure 6) suggests that these genes are likely relevant to cytosine methylation modification; the differential response to 5-AC among the lines might be due to their variable methylation patterns of these genes.

## Discussion

Hybridization is prevalent in plants, which plays important roles in genome evolution, and may lead to speciation at both the homoploid level and followed by genome doubling (allopolyploid) [6,7,33-37]. Apart from direct transfer and recombinatory generation of genetic variations by hybridization, *de novo *genetic and epigenetic instabilities can be induced by the process *per se*, including transcriptional activation and mobilization of cryptic transposable elements (TEs) [15,16,29,38-42]. Several lines of circumstantial evidence have indicated that introgression of DNA or chromatin fragments from an alien species into a recipient genome may also produce similar effects in causing genetic and epigenetic instabilities and generate novel phenotypes [11,17]. We have reported previously that introgressive hybridization between rice (*Oryza sativa*) and *Z. latifolia *had induced rampant mobilization of two TEs, a *copia*-like LTR retrotransposon *Tos17 *and a MITE *mPing *[21,43]. In this study, we extended the earlier findings and found that the *Dart*-related TEs were also transpositionally reactivated in the introgressants, although the elements were totally quiescent in the parental rice cultivar Matsumae. We validated the excisions and insertions by transposon-display (TEs) and sequencing, which ruled out genomic rearrangements as the major cause for the dramatically altered hybridization patterns detected by Southern blotting in the introgressants.

Although numerous studies have established correlative or causal links between TE activation and alteration in the element's cytosine methylation state [26,27,30,44,45], we found that the *Dart*-related TEs were similarly hypermethylated along their entire length in the introgressants and their rice parental line Matsumae. Nonetheless, moderate alteration in the methylation patterns was discernible in the introgressants. Furthermore, given that the introgressants were at the 9^th^-selfed generation and stabilized in both phenotype and DNA fingerprinting patterns [20], we could not rule out the possibility that in earlier generations of the introgressants (no longer available for study), more marked methylation remodelling might have occurred in the *Dart-*related TEs, which however were either largely reverted to the original pattern and/or those individuals with more drastically altered patterns had been purged out during the sexual reproduction, probably due to reduced fitness. Therefore, it remains a formal possibility that alteration in cytosine methylation had been associated with mobility of the *Dart*-related TEs in the introgressants, a scenario gaining increased empirical support in a vast range of TEs and organisms [9-11,26,28,30,46].

An array of studies in both plants and animals has established that activity of TEs, particularly LTR retrotransposons, may significantly impact expression and function of their adjacent genes [38,41]. Nonetheless, other studies have indicated that the majority of newly transposed TEs particularly those with small-sizes like MITEs tended to insert into functionally neutral genomic regions and impose minor effects on their adjacent genes [31]. We found in this study that the six single-copy protein-coding genes adjacent to the newly excised or inserted *Dart*-related TEs exhibited significantly altered expression in the introgressants relative to their rice parental line under both normal and several abiotic-stress conditions. However, the altered gene expression in the introgressants was not coupled with the TE excisions or insertions, suggesting that other regulatory mechanisms were responsible for the altered gene expression in the introgressants. Because unbiased amplifications between the introgressants and their rice parental line were observed when their genomic DNA was used as templates, it is likely that epigenetic regulation was involved. The observation that for most of the genes, these rice lines exhibited sharply differential response to 5-AC treatment corroborated this possibility, which also accords with our previous results showing that substantial re-patterning of cytosine methylation occurred in the introgressants for amny genomic loci [47]. Further study is required to elucidate the exact molecular basis underlying the dramatically altered gene expression and their phenotypic consequence in these novel rice lines as a result of introgressive hybridization.

## Conclusion

Results of this study have extended our previous findings by documenting that introgressive hybridization between rice and *Z. latifolia *has induced transpositional reactivation of another distinct family of cryptic TEs in the parental rice genome, namely, the *Dart*-related TEs, suggesting that introgression of chromatin from a realted alien species might have caused a general breakdown of the host cellular machinery responsible for repressive control of TE activity. Transposition of the *Dart*-related TEs was accompanied with a moderate alteration in the element's cytosine methylation in the introgressants. In addition, results of this study showed that extensive alteration in expression of a set of *Dart*-adjacent, protein-coding genes occurred in the introgressants relative to their rice parental line, under both normal and various abiotic stress conditions. Nonetheless, the alteration in gene expression was not coupled with excision or insertion of the *Dart*-related TEs, implicating other regulatory mechanism(s) was underpinning the changes in gene expression in these novel rice introgressants.

## Methods

### Plant lines

Three introgression lines (RZ1, RZ2 and RZ35) derived from a cross between rice (cv. Matsumae) and *Zizania latifolia *Griseb, were used in this study [21]. The three stabilized introgressants (at the 9^th ^selfed generation) were homogeneous in phenotype and DNA fingerprinting patterns, and exhibited heritable, novel morphological characteristics in multiple traits compared with their rice parental cultivar Matsumae [20,21]. The introgressants were maintained along with their rice parental line (cv. Matsumae) by strict selfing in our laboratory.

### Abiotic stress and 5-azacytidine treatments

Healthy and uniform seeds of three rice-*Zizania *introgressants (RZ1, RZ2 and RZ35) and their rice parental line cv. Matsumae were disinfected and thoroughly rinsed, and placed on petri-dishes covered with half-strength Murashige and Skoog (MS) medium in darkness at 25°C. For the three kinds of abiotic stress treatments, seedlings were grown to the 3-leaf-stage, and then aqueous solutions respectively containing 5 mM CuSO_4 _(heavy metal), 5 mM HgCl_2 _(heavy metal), 10 mM NaCl (salinity) and 10 mM NaHCO3 (alkaline) containing the half-strength MS medium were added, and grown for one more week. For cold stress, the 3-leaf-stage seedlings were grown in the medium at 12°C for one week. The 5-azacytidine (5-AC) treatment was conducted by treating the germinating seeds in the medium containing 50 mM 5-azacytidine (Sigma) for one week and then thoroughly rinsed with ddH_2_O and allowed the seedlings to grow in the medium up to the same stage as the other treatments. In all cases mock-control seedlings grown in the half-strength MS medium alone was included.

### Southern blot analysis

Genomic DNA was isolated from leaf tissue of young seedlings at the same developmental stage from the various treatments and mock of the three rice-*Zizania *introgressants (RZ1, RZ2 and RZ35) and their rice parental line Matsumae by a modified CTAB method. To assess possible genetic changes in the patterns of the *Dart*-related TEs, the genomic DNA (~3 μg, per lane) of each line (the mock-control) was digested by *Hin*dIII. To test for possible alteration in cytosine methylation of the *Dart*-related TEs, the genomic DNA of each line (the mock-control) was first digested with *Bam*HI (to delineate the *Dart*-related TEs into three regions, 5'-, 3'-, and body-regions; see *Results*), followed by a second round of digestion with a pair of isoschizomers, *Hpa*II and *Msp*I, that recognize the same sequence 5'-CCGG but with differential sensitivity to methylation of the two cytosine residues. Digested DNA were run on 1% agarose gel and transferred onto Hybond N+ nylon membrane (RPN 303B, Amersham-Pharmacia Biotech, Piscataway, New Jersey) by the alkaline transfer method recommended by the manufacturer.

In total, three pairs of primers specific respectively to the 5'-, 3'- and the body-region of the *Dart-*related TEs were designed to amplify the fragments to be used as hybridization probes. The primers are: (1) for the 5'-region of *Dart*-related TEs: *Dart*5'-forward: 5'- aaatagggcatgaaccccagc, *Dart*5'-reverse: 5'-ggtcgaaatcacccaaggtg; (2) for the 3'-region of *Dart*-related TEs: *Dart*3'-forward: 5'-tccagaccaaccccagtagaa, *Dart*3'-reverse: 5'-aaaaaaagcaaaggaaatgtataagg; (3) for the body-region of *Dart*-related TEs: *Dart*-body-forward: 5'-ctagagaggattatcttagcgtagttgtt, *Dart*-body-reverse: 5'-cttcttcttacctgtagtggggatag. Authenticity of the amplified fragments was verified by sequencing. The fragments were then agarose gel-purified and labelled with fluorescein-11-dUTP by the Gene Images random prime-labelling module (Amersham-Pharmacia Biotech). Hybridizations were done with the Gene Images CDP-Star detection module (Amersham-Pharmacia Biotech). After washing twice with 0.2×SSC, 0.1% SDS for 50 min. The filters were exposed to X-ray films for chemiluminescence signal detection.

### Transposon display (TD), locus-specific PCR amplification and sequencing

The genomic DNA (approximately 300 ng per sample) was cut with *Mse*I and subject to transposons display (TD) following the protocol essentially as reported [48]. Three consecutively nested, sequence-specific primers targeting to the 3' end of *Dart*-related TEs were designed. Two specific primers (TDPrm1/TDPrm2 and *Mse*I+C/G) were respectively combined with the selective-amplification primers targeting the adapters, while the most external primer (TIR+N/*Mse*I+3) was used for further validation of the isolated TD bands. Detailed information concerning adapters and primers are listed in Additional files 1. The PCR amplification conditions and TD amplification products were resolved by PAGE and visualized by silver-staining [20]. Only clear and reproducible variant bands between two technical replications were considered as putative new insertions by the *Dart-*related TEs, and recovered for sequencing.

Based on the sequencing results by identifying the expected 3'-terminus of the *Dart-*related TEs, the contiguous flanking regions were extracted and used to query the Nipponbare genome sequence http://rgp.dna.affrc.go.jp by BlastN, and a set of locus-specific primers each being downstream of the *Dart *but compatible with the *Dart*-specific TD primers were designed with Primer 3 http://biocore.unl.edu/cgi-bin/primer3/primer3_www.cgi and those produced the expected results were listed in Tables 1 and 2. Each of these primers were then sequentially combined with the nested *Dart*-specific TD primers to reproduce the putative excisions and insertions identified by TD in the introgressant(s), and the amplicons were then sequenced for validation.

### RNA isolation and quantitative real-time-reverse transcriptase (RT)-PCR analysis

Total RNA was isolated from the same young leaf tissue as used for DNA isolation and also from the root tissue of the same seedlings of the various treated and mock lines, with the Trizol Reagent (Invitrogen) according to the manufacturer's protocol. The RNA was then treated with DNaseI (Invitrogen) to eliminate possible genomic DNA contamination before being reverse transcribed with the SuperScript RNase H- Reverse Transcriptase (Invitrogen).

The expression of genes adjacent to the newly excised or inserted *Dart*-related TEs in the introgressant(s) was studied by quantitative real-time-RT-PCR using gene-specific primers (see Additional file 2). The q-RT-PCR experiments were performed using a Roche LightCycler480 apparatus (Roche Inc.) according to the manufacturer's instruction and SYBR Premix Ex Taq (TOYOBO) as a DNA-specific fluorescent dye. The primers for the six studied genes were designed by the Primer 5 software (see Additional file 2). Expression of a rice β-actin gene (Genbank accession X79378) was used as internal control with the primer pairs 5'-atgccattctccgtctt and 5'-gctcctgctcgtagtc. The choice of the β-actin gene as the internal control was based on previous investigation showing that expression of this house-keeping gene between the tissues and under the various stress conditions was constant [49]. Conditions of q-RT-PCR were as reported [50].

## Authors' contributions

NW carried out major parts of the experiments, analyzed the data and drafted the manuscript. HW, HW, DZ, CL, YW, XO and ZD participated in all the experiments. BL designed the work and finalized the manuscript. All authors read and approved the final manuscript.

## Supplementary Material

Additional file 1 **Primers used in the transposon-display (TD) assay**. Click here for file

Additional file 2 **Gene-specific primers used in expression analysis by real-time qRT-PCR**. Click here for file
