# Supplementary material for: Transpositional reactivation of the Dart transposon family in rice lines derived from introgressive hybridization with Zizania latifolia
Source: BMC Plant Biol. 2010 Aug 26;10:190. doi: 10.1186/1471-2229-10-190 (PMC2956540; doi:10.1186/1471-2229-10-190)
Supplement: Additional file 1 — Primers used in the transposon-display (TD) assay. [file 1471-2229-10-190-S1.DOC]

**Additional file 1.** List of primers used in the transposson-display (TD) assay.

| Adapters and primers | Sequences (5`-3`) and specifications |
| --- | --- |
| Adapter |  |
| MseI adapter | 5'-GACGATGAGTCCTGAG-3' |
| Preselective primers |  |
| MseI-C | 5'-GATGAGTCCTGAGTAAC-3' |
| MseI-G | 5'-GATGAGTCCTGAGTAAG-3' |
| TDPrm1 | 5'-GAGATACGGACTTGCGGAAGGATGA-3' |
| TDPrm2 | 5'-CAAATTTSAACGGTCAAATAGCCGTTAGTG-3'  where S represents G to C |
| Selective primers |  |
| MseI-C *+AA* | 5'-GATGAGTCCTGAGTAACAA-3' |
| MseI-C *+AC* | 5'-GATGAGTCCTGAGTAACAC-3' |
| MseI-C *+AG* | 5'-GATGAGTCCTGAGTAACAG-3' |
| MseI-C+ *AT* | 5'-GATGAGTCCTGAGTAACAT-3' |
| MseI-C *+TA* | 5'-GATGAGTCCTGAGTAACTA-3' |
| MseI-C *+TC* | 5'-GATGAGTCCTGAGTAACTC-3' |
| MseI-C *+TG* | 5'-GATGAGTCCTGAGTAACTG-3' |
| MseI-C *+TT* | 5'-GATGAGTCCTGAGTAACTT-3' |
| MseI-C *+CA* | 5'-GATGAGTCCTGAGTAACCA -3' |
| MseI-G *+AA* | 5'-GATGAGTCCTGAGTAAGAA-3' |
| MseI-G *+AC* | 5'-GATGAGTCCTGAGTAAGAC-3' |
| MseI-G *+AG* | 5'-GATGAGTCCTGAGTAAGAG-3' |
| MseI-G *+AT* | 5'-GATGAGTCCTGAGTAAGAT-3' |
| MseI-G *+TA* | 5'-GATGAGTCCTGAGTAAGTA-3' |
| MseI-G *+TC* | 5'-GATGAGTCCTGAGTAAGTC-3' |
| MseI-G *+TG* | 5'-GATGAGTCCTGAGTAAGTG-3' |
| MseI-G *+TT* | 5'-GATGAGTCCTGAGTAAGTT-3' |
| MseI-G *+CA* | 5'-GATGAGTCCTGAGTAAGCA -3' |
| TIR+N | 5'-GCCCRTTTGGCCACCTCTAN-3'  N represents A, C, G or T |
