# Supplementary material for: Transpositional reactivation of the Dart transposon family in rice lines derived from introgressive hybridization with Zizania latifolia
Source: BMC Plant Biol. 2010 Aug 26;10:190. doi: 10.1186/1471-2229-10-190 (PMC2956540; doi:10.1186/1471-2229-10-190)
Supplement: Additional file 2 — Gene-specific primers used in expression analysis by real-time qRT-PCR. [file 1471-2229-10-190-S2.DOC]

**Additional file 2.** Gene-specific primers used in expression analysis by real-time qRT-PCR.

| Genes | Sequences (5`-3`) |
| --- | --- |
| *Gene 1* | For: 5'-AGCGTTAATCCGTGCCTTTG-3'  Rev: 5'-GTCCCGTTGCCTGTGAGAAT-3' |
| *Gene 2* | For: 5'-GCAGTTCAGATGCAGGAAGATTG-3'  Rev: 5'-TGGTCCAGATGAGGTCGTTGTC-3' |
| *Gene 3* | For: 5'-CAGCCAAGCCCAAGCAGAA-3'  Rev: 5'-TAGGCGGTACACGGCAAGAT-3' |
| *Gene 4* | For: 5'-TGCCAACCGTCCGGTCAA-3'  Rev: 5'-AGCGGTCCACGCAAACACG-3' |
| *Gene 5* | For: 5' GTGCCTCCACCACATTTATCTC 3'  Rev: 5' TTTCCTTCGTATTCCATCCTCA 3' |
| *Gene 6* | For: 5' GGGAATGTAACACTAGGAAGGGA 3'  Rev: 5' CAACGAAGAAGAATACGGGCA 3' |
